# Modeling EphB4-EphrinB2 protein–protein interaction using flexible docking of a short linear motif

**DOI:** 10.1186/s12938-017-0362-7

**Published:** 2017-08-18

**Authors:** Maciej Pawel Ciemny, Mateusz Kurcinski, Maciej Blaszczyk, Andrzej Kolinski, Sebastian Kmiecik

**Affiliations:** 1 Faculty of Chemistry, Biological and Chemical Research Centre, University of Warsaw, Żwirki i Wigury 101, 02-089 Warsaw, Poland; 2 Faculty of Physics, University of Warsaw, Pasteura 5, Warsaw, Poland

**Keywords:** Molecular docking, Flexible docking, Protein–peptide docking

## Abstract

**Background:**

Many protein–protein interactions are mediated by a short linear motif. Usually, amino acid sequences of those motifs are known or can be predicted. It is much harder to experimentally characterize or predict their structure in the bound form. In this work, we test a possibility of using flexible docking of a short linear motif to predict the interaction interface of the EphB4-EphrinB2 complex (a system extensively studied for its significance in tumor progression).

**Methods:**

In the modeling, we only use knowledge about the motif sequence and experimental structures of EphB4-EphrinB2 complex partners. The proposed protocol enables efficient modeling of significant conformational changes in the short linear motif fragment during molecular docking simulation. For the docking simulations, we use the CABS-dock method for docking fully flexible peptides to flexible protein receptors (available as a server at http://biocomp.chem.uw.edu.pl/CABSdock/). Based on the docking result, the protein–protein complex is reconstructed and refined.

**Results:**

Using this novel protocol, we obtained an accurate EphB4-EphrinB2 interaction model.

**Conclusions:**

The results show that the CABS-dock method may be useful as the primary docking tool in specific protein–protein docking cases similar to EphB4-EphrinB2 complex—that is, where a short linear motif fragment can be identified.

## Background

As much as 40% of protein–protein interactions (PPIs) in higher eukaryotes are mediated by short linear motifs [1] (SLiMs). This fact was first reported for motifs embedded in unstructured protein regions [2] and most research on this topic followed this route. Recent studies show that many interactions of the globular proteins are also mediated by short linear fragments localized at the interface [3]. SLiMs frequently contribute to the majority of complex binding energy; in the set of CAPRI targets (rounds 1–19) and protein–protein docking benchmark 3.0 discussed in [4], the PPI energy was dominated by such a single linear motif localized at the interface in more than half of the cases.

Even if the localization of the interaction site is known, the prediction of protein–protein complex structure is still a demanding task [5]. The main reason for this is the need to consistently sample a great number of possible arrangements of the subunits. Moreover, SLiMs are most often localized in intrinsically disordered regions of the proteins whose structure changes on binding. Therefore, protein–protein docking methods need to overcome the challenge of predicting (sometimes significant) conformational changes. Coarse-grained protein models are among the most successful approaches in this field [6, 7]. The best performing tools available as online servers, according to the recent CAPRI evaluation experiment results [8], include: ClusPro [9], LZerD [10], SwarmDock [11] and Haddock [12]. Most of the available protein–protein docking protocols use rigid body docking for initial screening of possible poses. After a set of possible structures is generated, they are refined locally. One of the major challenges in protein–protein docking is modeling interactions mediated by unstructured regions of the proteins (loops or intrinsically disordered regions). Such cases require accounting for large conformational changes of the interaction interface, which is usually beyond the reach of the classical protein–protein docking tools. This creates the need for novel approaches, allowing for increased flexibility of the system during docking, such as the one presented here.

We propose and test a new protocol for protein–protein docking based on the flexible docking of a SLiM fragment (peptide) to a protein receptor without using any information about the SLiMs structure or a binding site. To perform this step, we use the CABS-dock online docking server [13–15] (available at http://biocomp.chem.uw.edu.pl/CABSdock/) that employs an efficient peptide docking scheme. Various methods exist for peptide–peptide docking [1] and some of them are available as web servers, such as GalaxyPepDock [16], RosettaFlexPepDock [17] or PepSite2 [18]. Those methods require different input data, for example an initial peptide structure (being close to the binding site) [17] or an interaction template(s) [16]. The CABS-dock does not require the knowledge about the binding site, nor the template information. Moreover, the CABS-dock provides full flexibility of the peptide and significant flexibility of the protein receptor during the search for the binding site. The ability to handle significant flexibility of receptor structures distinguishes the CABS-dock from other global docking methods [18, 19]. The CABS-dock methodology has also been shown to be successful in folding and binding simulations of an intrinsically disordered peptide [20]. Those features and results make the CABS-dock method well suited for the initial-stage prediction of the SLiM binding mode and identification of PPI interface localization. This innovative yet simple approach is applied to protein–protein complex structure prediction of the extensively researched EphB4-EphrinB2 complex. This dimer is involved in a variety of physiological functions including patterning, cell attachment and motility [21–23]. Probably, the main reason for the increasing interest in this structure is its overexpression and dysregulation in many tumor cell lines [24] and the possible role in pathological angiogenesis and tumorigenesis [25]. Moreover, the overexpression frequently correlates with malignancy and the rate of tumor progression [26]. The knowledge of the PPI details of this complex may be used for novel drug design for anti-angiogenesis and anti-tumorigenesis therapies targeting the EphB4-EphrinB2 interaction. For example, an attempt has been made to predict a peptide inhibitor that mimics part of the PPI interface, thus competing with one of the proteins for the binding site [4]. With our method, starting from structures of the subunits and the SLiM sequence, we obtained high quality models of the EphB4-EphrinB2 complex.

## Methods

EphB4-EphrinB2 complex is a model protein–protein system in which the PPI is dominated by a single SLiM [4]. SLiM sequences in proteins may be identified with different approaches. Commonly, these fragments are well conserved patterns that may be found using bioinformatics tools [27–29]. It is also possible to derive this information from mutation experiments. To test the validity of our approach, we used the sequence of the 13-residue peptide motif (residues 116–128) of EphrinB2 identified in the work of London et al. [4] based on the interface screening for highest-affinity linear segments. The SLiM in this complex is responsible for over 65% of the total interaction energy and is bound to a well-defined binding site [4]. Those features, combined with medical significance of the complex, make it an interesting test case for our proof-of-concept study.

**Fig. 1:**
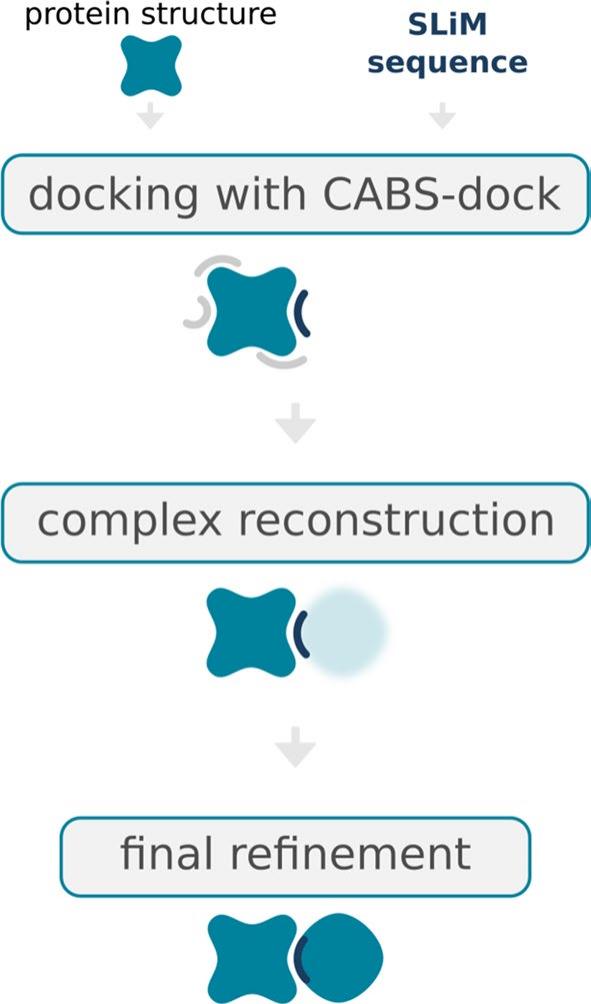
The protein–protein complex structure prediction pipeline. The *figure* shows three consecutive steps of our method: (*1*) CABS-dock based docking of the SLiM sequence to the protein (this step requires input protein structure and the SLiM sequence), (*2*) reconstruction of the complex based on CABS-dock prediction, and (*3*) final refinement of the complex

The protocol for EphB4-EphrinB2 protein–protein docking consist of the three steps presented in Fig. 1:

1. Using the identified motif sequence as the sequence of a peptide in flexible protein–peptide docking with the CABS-dock server [13–15];
2. Adjustment of the protein–protein complex based on the results of protein–peptide docking;
3. Refinement of the protein–protein complex using two methods: the FG-MD [30] and the GalaxyRefine [31] for a molecular dynamics based algorithm to atomic-level protein structure refinement. Both methods were used with their default settings.

In the first and crucial modeling step, the SLiM sequence is docked to the receptor protein structure in the bound form (PDB code 2HLE, chain A) using the CABS-dock server [13–15]. During docking, the SLiM is treated as a fully flexible peptide and it is allowed to search for the optimal binding site and pose over the entire receptor surface. Presently, the CABS-dock method belongs to the most efficient tools for flexible protein–peptide docking that enables large conformational changes during explicit docking [32]. This capability is possible thanks to the efficient CABS coarse-grained simulation scheme merged with all-atom modeling in the CABS-dock protocol. Apart from prediction of the protein–peptide complexes, CABS coarse-grained simulations have been successfully used in the modeling of protein interactions [20, 33, 34], folding mechanisms [35–37], structure flexibility [38, 39] and structure prediction [40, 41]. CABS model design and applications have been recently described in the review [6]. In a nutshell, CABS uses a coarse-grained protein representation in which each amino acid is represented by up to four pseudo-atoms: alpha carbon (CA), beta carbon (B), united side chain (S) and center of the peptide bond. CABS force field is statistical, derived from statistics of known protein structures. The force-field takes into consideration various regularities of local packing and secondary structure [42]. CABS sampling is controlled by the Replica Exchange Monte Carlo (REMC) scheme. CABS-dock docking procedure consists of the following steps (described in detail in works [13–15]):

- the receptor structure is converted into the CABS coarse-grained representation;
- an ensemble of 10 (one per replica for the REMC method) random peptide conformations in coarse-grained representation is generated;
- peptide replicas are placed at random locations around the receptor at the distance of 20 Å from the receptor’s surface;
- docking simulation is run with the completely flexible peptide and the receptor restrained to near-native conformation by a set of distance constraints derived from the starting conformation, a set of 10,000 models is generated (1000 per replica);
- 100 lowest energy states from each replica are selected for further processing;
- conformations of all 1000 (10 × 100) models are structurally clustered using the k-medoids procedure with L-RMSD (RMSD calculated on the peptide’s CA atoms after superposition of the receptor molecule) as the distance measure between them;
- 10 top-scored models, representatives of 10 most dense clusters (density defined as the number of cluster elements divided by average L-RMSD between them), are reconstructed to all-atom representation and refined in a short simulation in Modeller [43].

In the second step, a model of the complex is built. To do so, we use structures of both of the proteins, as well as information about the bound SLiM obtained with CABS-dock. In our study, we use the best binding pose of the SLiM peptide from the 10 top scored models provided by the CABS-dock server (in other cases, even partial knowledge about the binding site may be used to select an appropriate model out of the 10 top-scored models). The complex conformation is produced by performing RMSD-minimizing superposition of the peptide and its SLiM counterpart in the protein.

Finally in the third step, the derived protein–protein complex model is refined using all-atom refinement procedures [30, 44].

To analyze the applicability of our approach to the PPI interface prediction we use the interface-RMSD measure (iRMSD), which is calculated as the RMSD of the C-alpha atoms of the residues forming the SLiM interface. The interface is defined as the peptide together with the receptor residues within the 4.5 Angstroms cut-off (calculated based on positions of all heavy atoms).

Another way of assessing the predicted structure is analyzing the fraction of the native contacts (fNC) that were correctly predicted in the model. fNC values are calculated as the number of correctly predicted native contacts present in the model structure divided by the total number of native contacts. The contacts were calculated with the COCOMAPS Tool [45] with default settings (cut-off distance of 8 Å for contacts definition). This approach is perhaps more informative about prediction usefulness than iRMSD values.

## Results and discussion

**Fig. 2:**
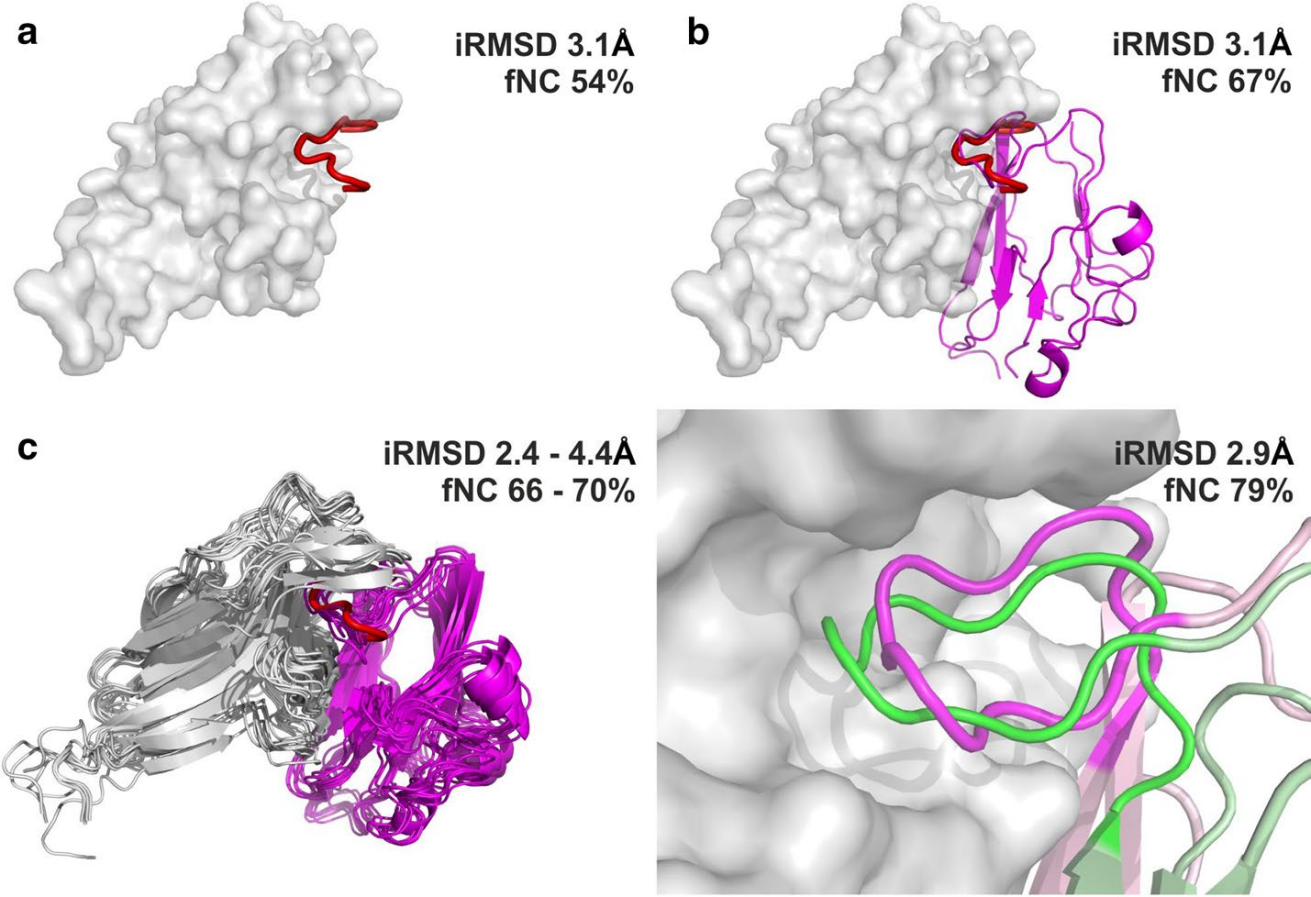
Visualization of the structures resulting from each of the modeling steps of the EphB4-EphrinB2 protein–protein interaction. For each modeling step interface RMSD (iRMSD) and fraction of native contacts (fNC) are provided. **a** Result of SLiM (from EphrinB2) docking to the EphB4 receptor (the SLiM is marked in *red*, EphB4 is visualized as a *gray surface*), **b** superimposition of the EphrinB2 structure on the docked SLiM peptide (the SLiM is marked in *red*, EphB4 is visualized as a *gray surface*, the structure of EphrinB2 is colored in *magenta*), **c** results of the complex refinement. The *left panel* shows the set of 10 models from the GalaxyRefine procedure. The *right panel* focuses on the interaction interface of the EphrinB2 SLiM (*magenta*) and EphB4 (*gray surface*) obtained from the FG-MD refinement procedure and its comparison with the experimental complex structure (PDB ID: 2HLE, shown in *green*)

**Fig. 3:**
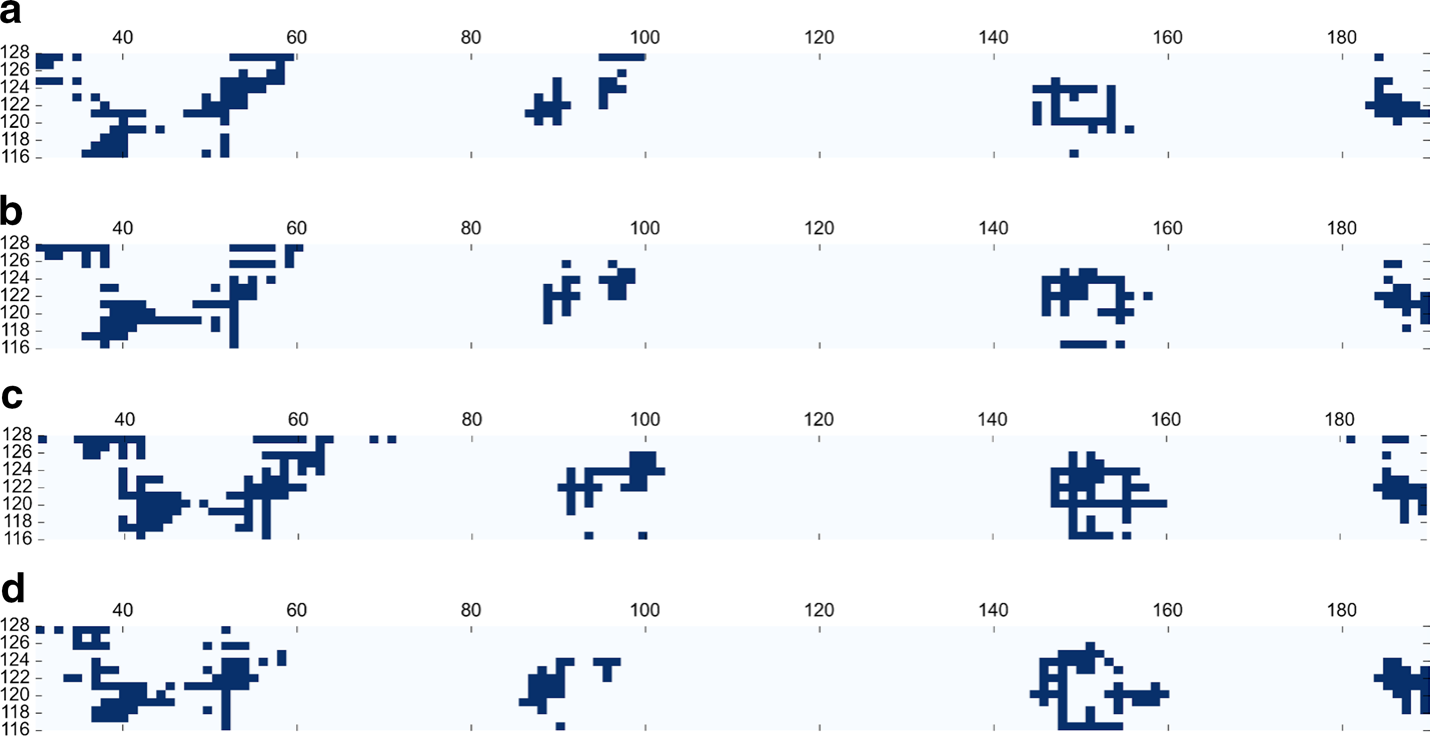
Comparison of contact maps at different modeling stages and the experimental complex structure for EphB4-EphrinB2 interaction. The *figures* show maps of contacts formed between the SLiM fragment localized on EphrinB2 and the protein EphB4 receptor for: **a** CABS-dock prediction, **b** superimposition, **c** final refinement, and **d** a reference map for the experimental complex structure (PDB ID: 2HLE). The maps were calculated with COCOMAPS tool [45] with default settings (cut-off distance of 8 Å)

We identified a pose with iRMSD value of 3.1 Å bound in the proximity of the native PPI interface (presented in Fig. 2a) among the top-scored models obtained with CABS-dock. The map of contacts between the predicted SLiM pose and EphB4 protein (presented in Fig. 3a) shows that it closely follows the native contact pattern (shown in Fig. 3d). Analysis of the map indicates that this pose reproduced as much as 54% native interactions in the SLiM region.

The model of the dimer obtained from superposition of the protein on the CABS-dock predicted SLiM conformation is presented in Fig. 2b. The iRMSD of the resulting structure was 3.1 Å. The contact pattern observed before is maintained and the fraction of correctly predicted native contacts for the SLiM region at this stage increased to 68%. The improvement in the quality of prediction in this step results from replacing the loose ends of SLiM CABS-dock prediction by the well-structured regions of the EphrinB2 protein.

The refinement performed with the GALAXY server resulted in a set of structures with iRMSD values in range 2.4–4.4 Å and the respective fNC of 66–70%. The FG-MD refinement procedure mostly improved the interface side-chains arrangement and produced a structure characterized by iRMSD of 2.9 Å. In consequence, the fraction of correctly predicted native contacts in the SLiM region in the final model further increased to 79%. The refined EphB4-EphrinB2 complex structure is presented in Fig. 2c.

Interestingly, even though the SLiM-based approach enables accurate prediction of the SLiM interface, the predicted orientation of the interacting domains is twisted by several degrees in respect to the native complex. As the SLiM fragment is localized in the flexible loop that could serve as a hinge between the subunits, one of them could be possibly rotated without PPI interface distortion but with significant improvement of the overall complex geometry. Unfortunately, modeling of such a large-scale conformational changes still remains a challenging task.

Further advances of our protocol are possible, including the incorporation of:

- Information about the binding site in the modeling process (taken from experiment or predicted using bioinformatics tools [27–29, 46]). Such data could be used as additional restraints in the CABS-dock docking simulations as well as during the refinement stage;
- An improved method for complex reconstruction from the docked SLiM pose. The RMSD-minimizing superposition we use here could be replaced with a method that performs rigid-body docking guided by the SLiM pose that could attempt to generate acceptable structures (as many variants as possible) for the further scoring and refinement step.
- An improved refinement method that could include: large-scale domain movements (this would allow more effective sampling of possible domain arrangements in cases when the PPI interface may serve as a hinge) and small-scale and large-scale backbone movements at the complex interface (allowing significant repacking of the complex interface).

## Conclusions

In this work, we used the CABS-dock method to predict the binding site and pose of the fragment of EphB4-EphrinB2 protein–protein complex, which allowed further reconstruction of the complex. The results we presented show that CABS-dock peptide (SLiM) docking may be a useful tool for protein–protein docking. The presented protein–protein docking scheme, applied here to modeling the EphB4-EphrinB2 interaction (see Fig. 1), can be easily modified or combined with more sophisticated procedures for computation modeling of protein interactions [47].
